# Liver Graft Proteomics Reveals Potential Incipient Mechanisms behind Early Renal Dysfunction after Liver Transplantation

**DOI:** 10.3390/ijms231911929

**Published:** 2022-10-08

**Authors:** Åsa Norén, Mihai Oltean, Styrbjörn Friman, Antonio Molinaro, Johan Mölne, Carina Sihlbom, Gustaf Herlenius, Annika Thorsell

**Affiliations:** 1 The Transplant Institute, Sahlgrenska University Hospital, 41345 Gothenburg, Sweden; 2 Department of Surgery, Institute of Clinical Sciences, Sahlgrenska Academy at the University of Gothenburg, 41345 Gothenburg, Sweden; 3 Wallenberg Laboratory, Department of Molecular and Clinical Medicine, Sahlgrenska Academy at the University of Gothenburg, 41345 Gothenburg, Sweden; 4 Department of Laboratory Medicine, Institute of Biomedicine, Sahlgrenska Academy at the University of Gothenburg, 40530 Gothenburg, Sweden; 5 Proteomics Core Facility, Sahlgrenska Academy at the University of Gothenburg, Medicinaregatan 5, 41390 Gothenburg, Sweden

**Keywords:** liver transplantation, ischemia–reperfusion injury, renal failure, proteomics, outcome

## Abstract

Acute kidney injury (AKI) is frequent after liver transplantation (LT) and correlates with later development of chronic kidney disease. Its etiology is multifactorial and combines pre-, intra-, and postoperative factors. Additionally, the liver graft itself seems an important element in the development of AKI, yet the detailed mechanisms remain unclear. We hypothesized that grafts of LT recipients developing significant early AKI may show distinct proteomic alterations, and we set out to identify proteome differences between LT recipients developing moderate or severe AKI (*n* = 7) and LT recipients without early renal injury (*n* = 7). Liver biopsies obtained one hour after reperfusion were assessed histologically and using quantitative proteomics. Several cytokines and serum amyloid A2 (SAA2) were analyzed in serum samples obtained preoperatively, 2–4 h, and 20–24 h after graft reperfusion, respectively. LT induced mild histological alterations without significant differences between groups but uniformly altered liver function tests peaking on postoperative day 1, with a trend towards more severe alterations in patients developing AKI. Global quantitative proteomic analysis revealed 136 proteins differing significantly in their expression levels (*p* < 0.05, FC 20%): 80 proteins had higher and 56 had lower levels in the AKI group. Most of these proteins were related to immune and inflammatory responses, host defense, and neutrophil degranulation. No differences between the studied pro- and anti-inflammatory cytokines or SAA2 between groups were found at any moment. Our results suggest that grafts of LT patients who develop early AKI reveal a distinct proteome dominated by an early yet prominent activation of the innate immunity. These findings support the hypothesis that AKI after LT may be favored by certain graft characteristics.

## 1. Introduction

Early results after liver transplantation (LT) have steadily improved over the last decades, with one-year patient and graft survival frequently exceeding 90% [1,2]. However, long-term outcome does not follow the same trend, and only about 60% of patients are alive ten years after the transplant [3]. The causes behind this attrition are multiple, and include disease recurrence, cardiovascular complications, diabetes, malignancies, or chronic renal failure [4]. The occurrence of chronic renal failure among patients with a nonrenal transplant is associated with an increase by a factor of more than four in the risk of death [5].

Renal dysfunction may occur at any time after LT. Acute kidney injury (AKI) has been reported in up to 95% of LT [6], and is strongly associated with the development of chronic kidney disease (CKD). The etiology of early AKI is multifactorial and combines pre-transplant (i.e., hepatorenal syndrome), intraoperative (bleeding, hemodynamic instability, post-reperfusion syndrome, medications) and postoperative risk factors (drug toxicity, infections). As the increasing use of livers from extended criteria donors has been paralleled by an increased incidence of early renal injury, the liver graft itself has emerged as an important element in the development of AKI [7,8]. Recent studies indicate that renal metabolism and function are significantly altered only hours after graft reperfusion in the absence of clear hemodynamic or pharmacologic causes [9]. Hence, the mechanisms through which the reperfused liver promotes the remote organ injury remain elusive.

Growing evidence suggests a causative role for liver-related factors, including an advanced hepatic ischemia reperfusion injury (HIRI) [10]. However, routine markers of liver injury such as transaminases and even microscopic examination regularly fail to discriminate between liver recipients who are going to develop AKI or not. Recent advancements in proteomics have allowed detailed insights into multiple biological processes at a previously unattained depth [11]. Using quantitative proteomics, we set out to identify alteration patterns in the livers of patients developing severe AKI after graft reperfusion. We hypothesized that certain liver grafts may show distinct molecular characteristics that may be related to the cascade of events ultimately leading to an impairment in renal function.

## 2. Results

### 2.1. Clinical Outcomes

One out of 27 patients initially included in the study required early retransplantation due to primary nonfunction, whereas the remaining 26 patients recovered and showed adequate postoperative liver graft function. Five patients developed AKI stage 1, eleven patients developed AKI stage 2 and 3, whereas the renal function of eleven patients remained unaffected by the LT (AKI stage 0). Liver biopsies were not available in seven patients. Hence, 14 patients (seven patients with and seven without AKI) and their corresponding biopsies form the basis of this report. The main donor and recipient characteristics for these 14 transplants are detailed in Table 1. Most variables were similar in both groups, with the exception of body mass index (BMI) both for the donors and recipients (see Table 1).

### 2.2. Liver Injury

**Figure 1:**
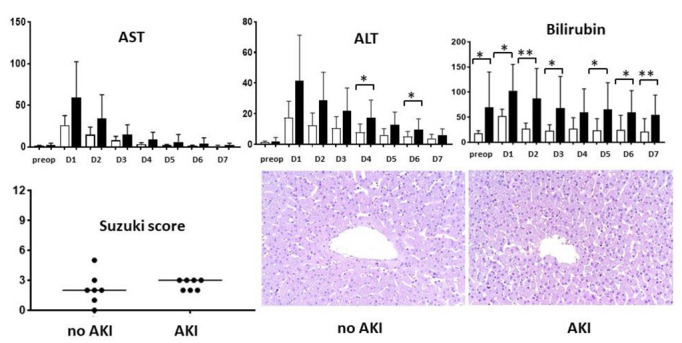
Biochemical and histological assessment of the ischemia–reperfusion injury. Daily liver function tests over the first week in patients without (white bars) and with acute kidney injury (black bars), semiquantitative assessment of the histology (Suzuki score), and representative microphotographs (hematoxylin–eosin, original magnification ×200). AKI: acute kidney injury; ALT: alanine aminotransferase; AST: aspartate aminotransferase; D: day. * *p* < 0.05, ** *p* < 0.01.

Liver graft preservation and reperfusion resulted in uniformly altered liver function tests peaking on postoperative day 1, without any significant difference between patients ultimately developing AKI or not (Figure 1). Likewise, HIRI induced only mild liver histological alterations without any significant difference between the histopathological Suzuki score in the two patient groups.

### 2.3. Proteomic Analysis

In the global quantitative biopsy analysis, a total of 4544 proteins were identified, and 3691 proteins were quantified. Comparing liver graft biopsies from patients with moderate/severe AKI with those from patients without kidney impairment, we found that 136 proteins displayed significant differences in their tissue expression (*p* < 0.05, FC ≥ 20%); 80 were upregulated and 56 downregulated in the patients developing moderate/severe AKI. Most of these proteins were related to immune and inflammatory responses, host defense, and neutrophil degranulation. The list of significantly regulated proteins, together with fold changes, corresponding *p* values, and relevant biological processes, are shown in Table 2.

**Figure 2:**
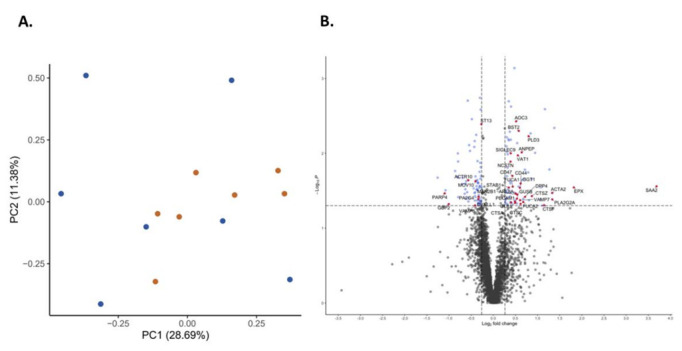
Principal component analysis showing data distribution in patients with acute kidney injury (blue) and without renal impairment (red) (**A**) and volcano plot indicating the proteins (red dots) showing both the magnitude of fold changes (*x* axis) and high statistical significance (-log10 of *p* values, *y* axis) (**B**).

For an overall assessment of proteomic similarities and differences between patients developing moderate/severe AKI with those from patients without kidney impairment, we employed PCA (Figure 2A). The PCA demonstrates that there were small differences between the two groups. Patients without AKI tended to be more similar to each other compared to the more heterogeneous group developing AKI 2 and 3.

Proteins associated with host defense, neutrophil degranulation, immune, and inflammatory responses are indicated in red in the volcano plot (Figure 2B). Several known inflammatory markers or enzymes such as SAA2 and PLA2 were among the most upregulated proteins in the group developing AKI.

**Figure 3:**
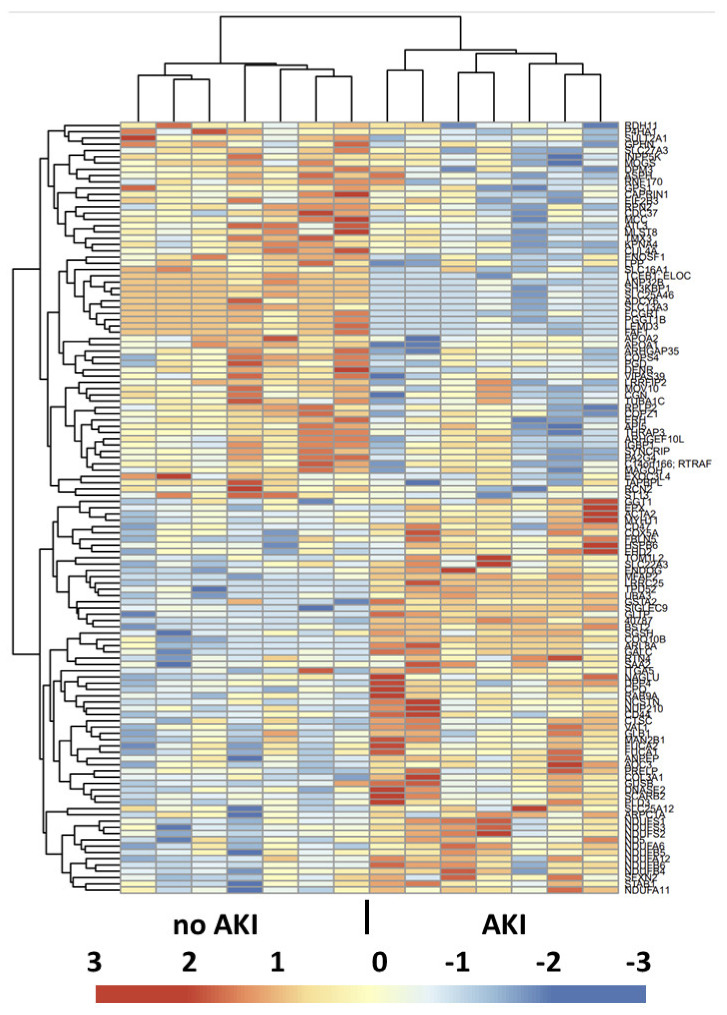
Heat map view and hierarchical clustering of the proteins showing significant differences between the two groups. The horizontal tree indicates the proteins, and the vertical tree indicates the 14 patients analyzed. The color scheme in the cluster analysis is from blue (low) to red (high), and protein identities are listed on the right.

For a functional view of the proteomic differences between the groups, hierarchical clustering of the statistically different expressed proteins was performed (Figure 3). The heat map clearly revealed the clustering of the various proteins differentially expressed between the two groups and allowed a distinct delineation of the two patient groups.

### 2.4. Validation Study—Immunofluorescence

**Figure 4:**
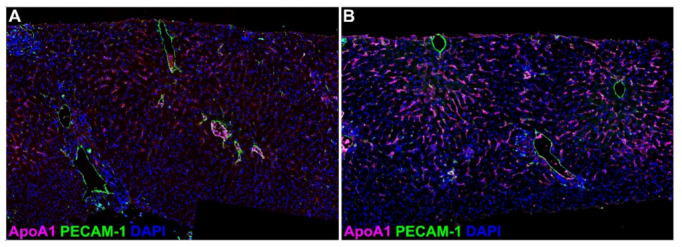
Detection of apolipoprotein A1 (pink, ApoA1) and platelet–endothelial cell adhesion molecule 1 (green, PECAM-1) by double immunofluorescence staining in postreperfusion liver biopsies. ApoA1 expression ranged from low (**A**) to high (**B**) and showed sinusoidal staining pattern; PECAM-1 expression was found on larger vessels (centrolobular and portal veins, arterioles). Nuclei were counterstained with DAPI (blue). Original magnification ×200.

Specific PECAM-1 and APOA1 staining was observed in all biopsies with variations in staining intensity and pattern (Figure 4). PECAM-1 staining was regularly found on the portal and centrilobular vein endothelia, as well as on some arterioles. Focal staining in the sinusoids was observed in some biopsies. Apolipoprotein A1 staining was found mainly in the sinusoids, whereas the central veins were largely negative. There was a strong correlation (Spearman) between proteomics results and the semiquantitative staining assessment (rho = 0.775, *p* = 0.0008).

### 2.5. Circulating Cytokines and SAA2

**Figure 5:**
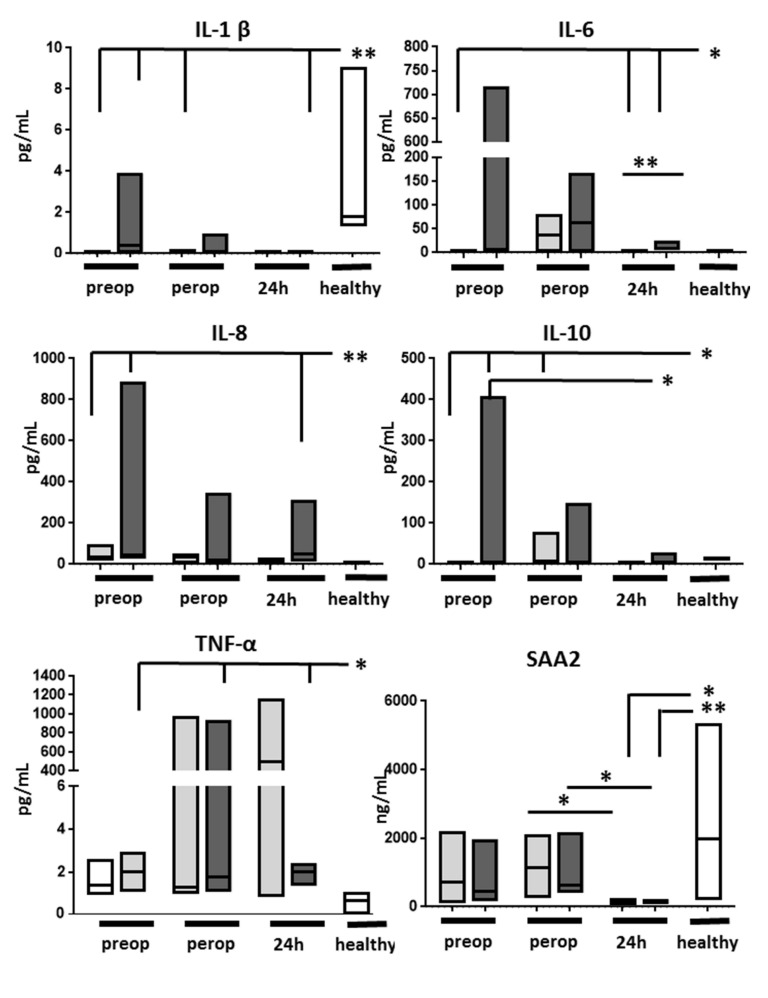
Serum concentration of several cytokines and serum amyloid A2 (SAA2) in patients without (light grey bars) and with acute kidney injury (dark grey bars) and in a group of healthy controls (white bars, *n* = 6). IL—interleukin, IFN—interferon. Lowest limits of detection were as follows: IFN-γ: 0.37 pg/mL, IL-1β: 0.05 pg/mL, IL-2: 0.09 pg/mL, IL-4: 0.02 pg/mL, IL-6: 0.06 pg/mL, IL-8: 0.07 pg/mL, IL-10: 0.04 pg/mL, IL-12p70: 0.11 pg/mL, IL-13: 0.24 pg/mL, TNF-α: 0.04 pg/mL. * *p* < 0.05; ** *p* < 0.01.

Circulating concentrations of a panel of cytokines were analyzed after 2–4 h and 24 h from graft reperfusion and compared to preoperative levels for each group as well as against healthy individuals (Figure 5). The preoperative levels of several cytokines revealed significant differences compared to healthy individuals which may reflect the underlying liver disease. Patients developing AKI had higher IL-6 at 24 h compared with those without AKI. Cytokines levels were also compared at each time point between the two study groups, but no significant differences in concentration levels were found for any of the tested cytokines.

Preoperative SAA2 levels in LT recipients were similar to those found in healthy subjects. SAA2 remained apparently unaltered by graft reperfusion but its levels decreased significantly 24 h after reperfusion, regardless of the presence of AKI.

## 3. Discussion

Acute kidney injury is a frequent complication after LT with major implications on overall outcome. Traditionally, the etiology of early AKI has been ascribed to perioperative factors centered around intraoperative hemodynamic alterations and drug toxicity, although more recent evidence suggests that factors related to the liver graft itself may play a role in its development. The current study found a distinct proteomic pattern in the liver grafts of patients who would develop AKI in the immediate early postoperative course.

Five out of the first ten proteins showing higher expression in the AKI grafts are related to inflammation and its development. As all these proteins were already present in the graft early after reperfusion, it is likely that their expressions were more donor-related rather than caused by transplantation/reperfusion. Indeed, SAA2 and PLA-2 can be induced by proinflammatory cytokines such as interleukin-1b and TNF-a [12,13], both universally increased during brain death [14]. Hence, it is likely that the causes behind the differential expression of some of the proteins originate in preprocurement events.

Donor BMI was higher in the AKI grafts, and several grafts in the AKI group showed steatosis, pointing towards a role of altered lipid metabolism in the genesis of AKI after LT. Whereas the negative effect of donor steatosis on recipient and graft outcome is well known [15], a negative impact of donor steatosis on recipient kidneys is less clear. Steatosis increases susceptibility to ischemia/reperfusion injury and ultimately alters microcirculation [16], increases the mitochondrial oxidative injury, or exacerbates the innate immune response, including granulocyte and myeloid cell recruitment and cytokine release [17]. Although the early microscopic assessment did not detect overt differences in HIRI, more subtle molecular events could have evolved differently in lean grafts compared to fatty grafts, ultimately affecting the renal outcome.

The intricate interplay between lipid metabolism and inflammation likely involves numerous molecules and needs to be considered in terms of their coordinated actions. One such candidate molecule appears to be PLA 2, which was found at the top of the protein list showing increased expression in the AKI grafts. Phospholipid degradation is an important event in the development of HIRI [18], as phospholipid hydrolysis by phospholipase A2 causes membrane phospholipid breakdown, and releases free fatty acids including arachidonic acids and lysophospholipids, which serve as precursors of various inflammatory lipid derivatives [19]. Subsequently, arachidonic acid metabolism results in formation of reactive oxygen species and lipid peroxides, which in turn provide potent proinflammatory stimuli. Hence, we suggest that increased PLA2 expression in the liver grafts in the AKI group may have favored a more intense oxidative stress and, eventually, a more intense proinflammatory milieu.

Serum amyloid A2 had the largest fold change between the two study groups, likely signaling a differentially expressed local inflammatory response. At the same time, circulating SAA2 levels did not differ between groups and were within normal range. Upregulation of hepatic SAA2 protein synthesis during the acute phase response requires several hours and involves a synergistic combination of cytokines (IL-1, IL-6, and TNF-α) and glucocorticoids [20,21]. As several hours are needed for SAA2 synthesis, we speculate that the SAA2 increase was likely initiated in the donor. Interestingly, circulating SAA2 levels dropped significantly 24 h after reperfusion, suggesting either short-lived or weak stimuli or a negative regulation, pharmacologic or biological, occurring during the first 24 h after graft reperfusion. Additionally, circulating IL-6 (but no other proinflammatory cytokines) appeared higher in patients developing AKI, further supporting the hypothesis of an inflammatory mechanism behind the post-transplant AKI.

The global proteomic analysis identified the differential tissue expression of numerous proteins involved in neutrophil degranulation in the two groups. Neutrophil infiltration and degranulation are essential, well-known events during the inflammatory phase of the reperfusion injury. Several cathepsins, lysosomal proteases which primarily act as proteolytic enzymes involved in tissue remodeling [22], were among the proteins differing the most between groups. Besides neutrophil degranulation, cathepsins also regulate apoptosis, autophagy, and activation of hormones, with cathepsin C as an emblematic member of the cathepsin family. Interestingly, our study found three distinct cathepsins among the proteins differing most between the two groups. Whereas the exact significance and consequences of increased cathepsin C, F, and Z remain unclear, it is likely that they reflect the ongoing neutrophil infiltration and degranulation, multiple ongoing cleavage processes, and activation of various substrates following HIRI [23]. Although the lytic effects are most likely local, systemic secretion and effects on distant organs cannot be excluded [24]. The intense ongoing proteolytic activity is further supported by the increased expression of other proteases, such as dipeptidyl peptidase 4 (DPP4), aminopeptidase N (ANPEP), carboxypeptidase Q (CPQ), and hydrolases (N-sulphoglucosamine sulphohydrolase), in the grafts of patients developing AKI.

Although the microscopic examination of these early biopsies did not discern differences between the grafts of recipients developing AKI or not, the different trend in transaminase leak suggests different degrees of hepatocellular injury (i.e., HIRI) occurring in these two study groups. An earlier analysis found that peak AST was an independent risk factor for the development of AKI after liver transplantation, directly linking HIRI to renal injury [10]. This hypothesis is also supported by experimental evidence where plasma ALT and creatinine (and renal dysfunction) had a direct and linear relationship following murine HIRI [25]. Nonetheless, although transaminases can be informative regarding HIRI status, both AST and ALT levels initially increase manifold regardless of the severity of HIRI. A large clinical study where liver grafts were assessed both histologically and using routine liver function tests suggests instead that the drop in transaminases and its levels towards the end of the first post-transplant week could discriminate between patients with or without histologically proven HIRI [26]. If this holds true, the current results may actually suggest that liver graft recipients developing AKI may have in fact had a more advanced HIRI.

This dataset differs significantly from the limited available proteomics data in human livers undergoing ischemia–reperfusion injury or transplantation [27]. An explanation is the different study design and hypothesis but also the state-of-the-art proteomics used herein. An earlier study by Vascotto et al., using two-dimensional gel electrophoresis (2-DE) on liver graft samples, identified around 900 proteins, with 36 proteins differentially expressed during HIRI [28]. However, many of these proteins were found in as little as only one out of the nine paired samples, and the conclusions of the study were rather limited. Another similar study could identify 1580 proteins in total, with just about 140 proteins altered during reperfusion of the liver. Similarly to the study of Vascotto et al., only several proteins of these were consistently found in most of the studied grafts [29].

This analysis adds novel and comprehensive information on the proteome of liver grafts in patients ultimately developing AKI. This hypothesis has not been addressed so far as most studies focused on the development and analysis of HIRI [27]. A strength of this study is the high-performing global mass spectrometry used herein, allowing us to identify over four thousand distinct proteins and allowing its quantitative assessment. This is in contrast to the limited available data on HIRI using proteomics, which is mostly based on older proteomic strategies with low output and subject to technical errors. Another strength is the delayed introduction of tacrolimus which allowed us to exclude CNI nephrotoxicity as cause of early renal impairment. Besides that, tacrolimus has known modulating effects on reperfusion injury, which could have influenced multiple signaling pathways and biological processes [30,31].

The ongoing controversy regarding whether small postoperative increases in serum creatinine level (KDIGO grade 1) are due to AKI or not [32] led us to exclude patients developing mild kidney injury from the analysis, following their prospective inclusion. Apart from renal causes, serum creatinine can be affected by acute changes in creatinine production and/or sarcopenia as well as an altered volume of distribution. Hence, this exclusion was made in order to ensure that renal injury is the dominant cause behind the creatinine increase, obtaining a less heterogenous group as well as reducing the potential biologic variability.

This study is limited by the rather short observation time after graft reperfusion and by tissue sampling being performed at only one time point, which precluded in-depth analyses on the dynamics of certain proteins or pathways and advanced mechanistic hypotheses. It is likely that the molecular landscape will change dramatically over the first hours and days due to the intense transcriptional activity after graft reperfusion [33]. Indeed, a proteomic analysis of human livers undergoing cold storage and reperfusion revealed rapid alterations (both increases and decreases) of about 30 proteins (adaptors, kinases, GTPases) involved in signaling and cytoskeleton remodeling as soon as ten minutes after reperfusion, followed by further changes during the first hour of reperfusion [34]. Although the current data provide only a snapshot from a very complex and evolving process, they allow to delineate a group of grafts whose recipients will develop early renal complications. We find it likely and very interesting that the short time lapse from reperfusion until obtaining the biopsy did not allow for de novo protein synthesis, and, probably, at the same time, also limited the protein degradation due to oxidative stress and protease activation. Overall, we assume that many of the differences noted herein were due to initial differential expression in the donor, rather than protein degradation during cold storage and after reperfusion.

In conclusion, we found that grafts of LT patients who develop early AKI have a distinct proteome dominated by an early activation of the innate immunity, supporting the hypothesis that AKI after LT may be favored by intrinsic graft characteristics.

## 4. Materials and Methods

### 4.1. Patients and Study Design

The study protocol was reviewed and approved by the Regional Ethical Review Board in Gothenburg (Dnr: 598-13) and was conducted in accordance with the 2013 Declaration of Helsinki. Twenty-seven nonconsecutive patients undergoing primary liver transplantation between March 2014 and February 2015 were initially enrolled in the study. Donor livers were perfused and stored in either histidine–tryptophan–ketoglutarate solution (Custodiol, Fresenius, Bad Alsbach, Germany), Belzer-University of Wisconsin solution (Carnamedica, Warsaw, Poland) or Institute Georges Lopez-1 (IGL-1) (Institute Georges Lopez, Lissieu, France) solution, and kept in static cold storage until transplantation. A detailed description of all 27 donor and recipient characteristics, as well as the perioperative management, is presented elsewhere [35]. In short, liver transplantation was performed with the preservation of the recipient vena cava without the use of veno-venous bypass or portocaval shunts. Liver graft reperfusion was initiated after completion of the cavo-caval and portal vein anastomoses and before performing the arterial and biliary anastomoses. Cold ischemia time (CIT) was defined as duration from the start of cold perfusion in the donor to portal reperfusion in the recipient. Immunosuppression consisted of induction with intravenous basiliximab (day 0 and POD 4) and intraoperative corticosteroids. Maintenance immunosuppression consisted of mycophenolate mofetil introduced on day 0 and tacrolimus introduced on POD 3, with additional oral corticosteroids for patients with primary sclerosing cholangitis and autoimmune hepatitis.

A liver graft biopsy was obtained using a 14-gauge automated biopsy gun at the end of the transplant procedure, about 1 h after reperfusion. Biopsies were placed in buffered formalin until processing. A blood sample was obtained preoperatively, 2–4 h, and 20–24 h after graft reperfusion, respectively. Serum was recovered and stored in aliquots at −80 °C until analysis.

Patients were considered to have renal dysfunction if they presented AKI stage 2 and 3 according to Kidney Disease: Improving Global Outcome (KDIGO) criteria (see below) within the first 48 h after graft reperfusion. Patients without any evidence of renal dysfunction (stage 0) during the same timeframe formed a control group. Patients showing only mild renal dysfunction (stage 1) were excluded from the analyses.

### 4.2. Assessment of Organ Dysfunction

Daily liver function tests (AST, ALT, bilirubin, INR) were recorded over the first week as a surrogate marker of HIRI, whereas daily serum creatinine levels within the first week were used to evaluate early AKI. AKI was defined according to the KDIGO criteria and stages, without the inclusion of urine output in the creatinine-based formula [36]. The four stages are as follows: no AKI; AKI stage 1: rise in serum creatinine of 1.5–1.9 times baseline or an increase of ≥0.3 mg/dL within 48 h; stage 2: rise in serum creatinine of 2.0–2.9 times baseline; and stage 3: 3 times baseline or an increase in serum creatinine to ≥4.0 mg/dL or need for renal replacement therapy.

### 4.3. Histology

Formalin-fixed tissue was paraffinized, embedded, and cut into five-micron sections. Sections were stained with hematoxylin and eosin and assessed by an experienced transplant pathologist using the Suzuki score (Table 3) [37].

Immunofluorescence was used to confirm the results of the global proteomics analysis. We studied the expression of apolipoprotein A1 (ApoA1) and platelet–endothelial cell adhesion molecule (PECAM)-1 according to the Opal protocol (PerkinElmer/Akoya, Waltham, MA, USA) according to manufacturers’ instructions using primary antibodies against ApoA1 (#ab52945, Abcam, UK) and PECAM-1 (#ab281583, Abcam). Slides were then examined blindly by an experienced pathologist, and protein overall expression was evaluated semiquantitatively from weak (+) to strong (+++). The results were then correlated with the proteomics (Spearman).

### 4.4. Global Protein Quantification

Proteins were quantified relatively as previously described [38]. In short, proteins were extracted from the formalin-fixed paraffin embedded samples and digested using a modified filter aided sample preparation protocol. Peptide samples were chemically labeled with tandem-mass-tag (TMT, Thermo Fisher Scientific, Waltham, MA, USA) for relative quantification, and the 20 fractions for each set from basic reverse phase separation were analyzed with nanoLC on an Orbitrap Fusion Tribrid mass spectrometer (Thermo Fisher Scientific) operating in MultiNoch MS3 mode. Protein identification and quantification were performed with Proteome Discoverer version 2.4 (Thermo Fisher Scientific) matching against Swissprot Homo sapiens database (January 2021). Differential expression analysis using a two-sample *t*-test on log2-transformed data was performed using the Perseus software (1.6.15.0) and R. Proteins with a *p* value < 0.05 and fold change (FC) ≥ 20% were considered differentially expressed.

### 4.5. Cytokine and Serum Amyloid A2

Samples were analyzed for a panel of cytokines using the multiplex technique. Serum concentration of IFN-γ, IL-1β, IL-2, IL-4, IL-6, IL-8, IL-10, IL-12p70, IL-13, and TNF-α was determined by the electrochemiluminescence multiplex system Sector 2400 imager from Meso Scale Discovery (K15049D-1, Gaithersburg, MD, USA). Analytes below detection limit were inputted as half the lower limit of detection in order to facilitate statistical analysis.

SAA2 serum levels were evaluated in duplicate using an ELISA kit (LS-BIO, LS-F4984, Seattle, WA, USA) according to the manufacturer’s protocol.

### 4.6. Statistical Analysis

For the proteomic analysis, the differential expression analysis was performed using the Perseus software (1.6.15.0) and R. Differentially expressed proteins were identified by using a two-sample *t*-test on log-transformed data. Proteins with a *p* value < 0.05 and fold change ≥ 20% were considered differentially expressed. Principal component analysis (PCA) and heat maps were used as quality control for the samples and clustering of groups. Patient-related variables were analyzed using GraphPad Prism 5.0 (Graphpad, San Diego, CA, USA). Categorical variables were assessed using chi square test, whereas continuous variables were analyzed using nonparametric tests (Kruskal–Wallis test followed by Mann–Whitney test), due to the small sample size and distribution of the results. A *p* value less than 0.05 denoted statistical significance.

## Figures and Tables

**Table 1:** Donor and recipient characteristics. Data are given as *n* (%) or median (IQR). * Etiologies are not mutually exclusive. ** Polycystic liver disease (*n* = 1). Non-alcoholic fatty liver disease (*n* = 1). AKI: acute kidney injury; BMI: body mass index; ICU: intensive care unit; DRI: donor risk index; MELD: Model for End-stage Liver Disease; mGFR: measured glomerular filtration rate; RRT: renal replacement therapy.

	All (*n* = 14)	AKI 0 (*n* = 7)	AKI 2 + 3 (*n* = 7)	*p* Value
**Donor**				
Age, years	62 (33–67)	45 (25–71)	62 (59–64)	0.97
Gender: female/male	4/10	2/5	2/5	1
BMI	25 (23–29)	23 (18–28)	29 (24–30)	**0.04**
Cause of death				
Cerebrovascular accident	5	1	4	0.27
Trauma	6	5	1	0.10
Other	3	1	2	1
ICU stay, hours	36 (25–58)	36 (16–65)	36 (28–57)	1
Preservation solution: Custodiol/UW/IGL-1	9/3/2	6/1/0	3/2/2	0.34
DRI	1.8 (1.3–1.9)	1.4 (1.3–1.8)	1.9 (1.4–2.0)	**0.02**
Cold ischemia time, min	431 (374–625)	437 (375–637)	399 (372–621)	0.81
**Recipient**				
Age, years	44 (34–56)	41 (24–57)	49 (28–56)	1
Gender: female/male	2/12	1/6	1/6	1
BMI	25 (20–29)	20 (19–27)	29 (21–32)	**0.03**
Diabetes Mellitus	2	0	2	0.46
MELD score	12 (7–15)	8 (7–12)	15 (12–20)	**0.03**
Ascites	6	3	3	1
Etiology of liver disease *				
Primary sclerosing cholangitis	6	3	3	1
Alcohol	2	2	0	0.46
Hepatitis B virus	1	1	0	1
Hepatitis C virus	3	0	3	0.19
Hepatocellular carcinoma	4	1	3	0.56
Other **	2	1	1	
Duration of surgery, hours	6 (6–8)	6 (5.5–7.5)	8 (6–9.5)	0.27
Intraoperative bleeding, mL	1150 (575–2425)	650 (500–2500)	1800 (1000–2400)	0.33
Serum creatinine at admission, mg/dL	0.78 (0.64–1.0)	0.8 (0.71–1.0)	0.76 (0.62–1.03)	0.60
mGFR, ml/min/1.73 m²	102 (98–109)	99 (84–110)	102 (101–103)	0.52
Post-reperfusion syndrome	0			
ICU stay, hours	22 (15–50)	24 (16–54)	20 (14–49)	0.90
RRT	0			

**Table 2:** List of proteins differentially expressed in the liver grafts of patients developing early AKI compared to liver grafts of patients with uneventful course.

Accession	Gene Symbol	Description	Fold Changes (Average Value)	*p* Value	Biological Process	Biological Function
P0DJI9	SAA2	Serum amyloid A-2 protein	12.87	0.03	Inflammation	Acute-phase protein
P11678	EPX	Eosinophil peroxidase	3.53	0.03	Inflammation	Neutrophil degranulation
P02461	COL3A1	Collagen alpha-1(III) chain	2.60	<0.01	Cell structure	Assembly of collagen
P14555	PLA2G2A	Phospholipase A2, membrane associated	2.52	0.04	Inflammation	Antimicrobial peptide
P62736	ACTA2	Actin	2.51	0.03	Cell structure	Vascular smooth muscle contraction
Q14108	SCARB2	Lysosome membrane protein 2	2.41	0.02	Transport	Lysosome structure
P09210	GSTA2	Glutathione S-transferase A2	2.24	<0.01	Cell differentiation	Glutathione conjugation
Q9UBX1	CTSF	Cathepsin F	2.22	0.05	Inflammation	MHC-II antigen presentation
P41219	PRPH	Peripherin	2.15	0.05	Cell structure	Axonal regeneration after injury
P35749	MYH11	Myosin-11	2.11	0.03	Structural protein	Smooth muscle contraction
P21810	BGN	Biglycan	2.10	0.04	Cell structure	Articular cartilage development
P55327	TPD52	Tumor protein D52	1.92	0.01	Cell differentiation	Golgi associated vesicle biosynthesis
P51888	PRELP	Prolargin	1.83	0.02	Cell aging	Anchoring collagen I-II
Q9UBR2	CTSZ	Cathepsin Z	1.83	0.04	Inflammation	Proteolysis, metabolism of angiotensinogen to angiotensins, neutrophil degranulation
Q9Y646	CPQ	Carboxypeptidase Q	1.79	0.01	Metabolic process	Post-translation protein modification
O76070	SNCG	Gamma-synuclein	1.75	0.04	Cell-cell interaction	Neurofilament network integrity
Q8IV08	PLD3	5’-3’ exonuclease PLD3	1.74	0.01	Inflammation	Phagocytosis, synthesis of phosphatidylglycerol
Q9NVA2	SEPTIN11	Septin-11	1.73	0.02	Cell division	Bacterial invasion of epithelial cells
P27487	DPP4	Dipeptidyl peptidase 4	1.64	0.04	Metabolic process	Protein digestion and absorption GLP1
Q9UBX5	FBLN5	Fibulin-5	1.64	0.02	Cell structure	Elastic fibers associated protein
P54803	GALC	Galactocerebrosidase	1.63	0.02	Metabolic process	Glycosphingolipid metabolism
O00115	DNASE2	Deoxyribonuclease-2-alpha	1.60	0.04	Cell death	Lysosome components
Q9BTY2	FUCA2	Plasma alpha-L-fucosidase	1.60	0.04	Metabolic process	Regulation of insulin-like growth factor transport and uptake
P55001	MFAP2	Microfibrillar-associated protein 2	1.57	0.02	Cell structure	Elastic fibers associated protein
P15144	ANPEP	Aminopeptidase N	1.56	0.01	Angiogenesis	Metabolism of angiotensinogen to angiotensins, neutrophil degranulation
P16070	CD44	CD44 antigen	1.53	0.03	Cell-cell interaction. Inflammation.	Hyaluronan collagen interaction protein
P53634	CTSC	Dipeptidyl peptidase 1	1.53	0.05	Immune response	Chaperon binding
P19440	GGT1	Glutathione hydrolase 1 proenzyme	1.52	0.03	Metabolic process	Glutathione synthesis and recycling
P51809	VAMP7	Vesicle-associated membrane protein 7	1.52	0.04	Transport	Cargo recognition for clathrin-mediated endocytosis, Golgi associated vesicle biogenesis
Q10589	BST2	Bone marrow stromal antigen 2	1.49	0.01	Inflammation	Interferon alpha/beta signaling, neutrophil degranulation
O14558	HSPB6	Heat shock protein beta-6	1.48	0.03	Metabolic process	Smooth muscle vasorelaxation and cardiac myocyte contractility
Q99536	VAT1	Synaptic vesicle membrane protein VAT-1	1.47	0.01	Metabolic process	Neutrophil degranulation
O00754	MAN2B1	Lysosomal alpha-mannosidase	1.46	0.04	Metabolic process	Lysosomal oligosaccharide catabolism, neutrophil degranulation
P08236	GUSB	Beta-glucuronidase	1.45	0.04	Metabolic process	Degradation of dermatan and keratan sulphate
P51688	SGSH	N-sulphoglucosamine sulphohydrolase	1.45	0.01	Metabolic process	Heparan sulfate degradation
Q9H2V7	SPNS1	Protein spinster homolog 1	1.45	0.05	Transport	Sphingolipid transporter
Q16853	AOC3	Membrane primary amine oxidase	1.43	<0.01	Inflammation	Phase I functionalization of compounds
Q96BM9	ARL8A	ADP-ribosylation factor-like protein 8A	1.42	0.03	Cell division	Lysosome motility
P10619	CTSA	Lysosomal protective protein	1.41	0.05	Inflammation	Glycosphingolipid metabolism. Neutrophil degranulation
P16284	PECAM1	Platelet endothelial cell adhesion molecule	1.41	0.04	Inflammation	Leukocyte trans-endothelial migration
P54802	NAGLU	Alpha-N-acetylglucosaminidase	1.41	0.04	Metabolic process	Degradation of heparan sulphate
Q14249	ENDOG	Endonuclease G	1.39	<0.01	Cell aging	Apoptosis
O75751	SLC22A3	Solute carrier family 22 member 3	1.38	0.03	Transport	Abacavir transmembrane transport
P04066	FUCA1	Tissue alpha-L-fucosidase	1.36	0.03	Inflammation	Neutrophil degranulation
Q08722	CD47	Leukocyte surface antigen CD47	1.35	0.02	Cell-cell interaction. Inflammation	Modulation of integrins
O75746	SLC25A12	Calcium-binding mitochondrial carrier protein Aralar1	1.34	0.05	Transport	Epileptic encephalopathy
P16278	GLB1	Beta-galactosidase	1.32	0.05	Metabolic process	Galactose metabolism
Q86Y39	NDUFA11	NADH dehydrogenase [ubiquinone] 1 alpha subcomplex subunit 11	1.32	0.01	Metabolic process	Complex I biogenesis
Q8N386	LRRC25	Leucine-rich repeat-containing protein 25	1.32	<0.01	Inflammation	Interferon signaling pathway
P07203	GPX1	Glutathione peroxidase 1	1.31	0.04	Cell death	Detoxification of reactive oxygen species
Q92542	NCSTN	Nicastrin	1.31	0.01	Cell proliferation	Alzheimer’s disease, NOTCH signaling pathway
Q96NB2	SFXN2	Sideroflexin-2	1.31	0.03	Transport	Transport of serine into mitochondria
Q9NQC3	RTN4	Reticulon-4	1.31	0.03	Cell structure	Formation and stabilization of ER tubules
Q9NZD2	GLTP	Glycolipid transfer protein	1.31	0.02	Transport	Transfer of various glycosphingolipids
Q9Y336	SIGLEC9	Sialic acid-binding Ig-like lectin 9	1.31	0.01	Metabolic process	Sialic-acid dependent binding to cells
P08648	ITGA5	Integrin alpha-5	1.30	0.04	Cell differentiation	Interaction with fibronectin and fibrinogen
P20674	COX5A	Cytochrome c oxidase subunit 5A	1.29	0.01	Metabolic process	Oxidative phosphorylation
Q9NY15	STAB1	Stabilin-1	1.28	0.03	Cell-cell interaction	Scavenger receptor for acetylated low-density lipoprotein
P56556	NDUFA6	NADH dehydrogenase [ubiquinone] 1 alpha subcomplex subunit 6	1.27	<0.01	Metabolic process	NADH to the respiratory chain
Q8TBC4	UBA3	NEDD8-activating enzyme E1 catalytic subunit	1.27	0.01	Metabolic process	Antigen processing: ubiquitination and proteasome degradation
Q9H8M1	COQ10B	Coenzyme Q-binding protein COQ10 homolog B	1.27	0.01	Metabolic process	Respiratory electron transport
O43674	NDUFB5	NADH dehydrogenase [ubiquinone] 1 beta subcomplex subunit 5	1.25	0.03	Metabolic process	Mitochondrial membrane respiratory chain NADH dehydrogenase
O75306	NDUFS2	NADH dehydrogenase [ubiquinone] iron-sulfur protein 2	1.25	0.01	Metabolic process	NADH to the respiratory chain
P03915	ND5	NADH-ubiquinone oxidoreductase chain 5	1.25	0.02	Transport	Complex I biogenesis
Q9NZN4	EHD2	EH domain-containing protein 2	1.25	0.03	Transport	Endocytosis, internalization of GLUT4
P28331	NDUFS1	NADH-ubiquinone oxidoreductase 75 kDa subunit	1.24	0.01	Metabolic process	Mitochondrial membrane respiratory chain
P36969	GPX4	Phospholipid hydroperoxide glutathione peroxidase	1.24	0.05	Metabolic process	Glutathione metabolism
P51178	PLCD1	1-phosphatidylinositol 4,5-bisphosphate phosphodiesterase delta-1	1.24	0.04	Metabolic process	The production of the second messenger molecules
Q9UI09	NDUFA12	NADH dehydrogenase [ubiquinone] 1 alpha subcomplex subunit 12	1.24	0.02	Metabolic process	NADH to the respiratory chain
O75489	NDUFS3	NADH dehydrogenase [ubiquinone] iron-sulfur protein 3	1.23	0.01	Metabolic process	NADH to the respiratory chain
O95139	NDUFB6	NADH dehydrogenase [ubiquinone] 1 beta subcomplex subunit 6	1.23	0.01	Metabolic process	Mitochondrial membrane respiratory chain NADH dehydrogenase
O95168	NDUFB4	NADH dehydrogenase [ubiquinone] 1 beta subcomplex subunit 4	1.22	0.04	Transport	Mitochondrial membrane respiratory chain NADH dehydrogenase
Q6ZVM7	TOM1L2	TOM1-like protein 2	1.22	0.03	Transport	Protein transport, mitogenic signaling
P51151	RAB9A	Ras-related protein Rab-9A	1.21	0.04	Metabolic process	Trafficking of melanogenic enzymes
Q8TEM1	NUP210	Nuclear pore membrane glycoprotein 210	1.21	0.04	Metabolic process	RNA transport
Q92747	ARPC1A	Actin-related protein 2/3 complex subunit 1A	1.21	0.05	Cell structure	Mediates the formation of branched actin networks
O15162	PLSCR1	Phospholipid scramblase 1	1.20	0.02	Cell death	Lipid scrambling, lipid flip-flop
O43678	NDUFA2	NADH dehydrogenase [ubiquinone] 1 alpha subcomplex subunit 2	1.20	<0.01	Metabolic process	NADH to the respiratory chain
Q9HD45	TM9SF3	Transmembrane 9 superfamily member 3	1.20	0.03	Metabolic process	Unknown
P09669	COX6C	Cytochrome c oxidase subunit 6C	1.19	0.02	Metabolic process	Respiratory electron transport
Q96K19	RNF170	E3 ubiquitin-protein ligase RNF170	0.80	0.04	Metabolic process	Stimulus-induced inositol 1,4,5-trisphosphate receptor type 1 (ITPR1) ubiquitination and degradation via the endoplasmic reticulum-associated degradation (ERAD)
Q9BZZ5	API5	Apoptosis inhibitor 5	0.80	0.04	Cell death	Protein assembly
Q9P2X0	DPM3	Dolichol-phosphate mannosyltransferase subunit 3	0.80	0.03	Metabolic process	Stabilizer subunit of the dolichol-phosphate mannose (DPM) synthase complex
Q9UQ80	PA2G4	Proliferation-associated protein 2G4	0.80	0.04	Cell proliferation	Erbb3-regulated signal transduction pathway
P63167	DYNLL1	Dynein light chain 1	0.79	0.04	Transport	Cargos protein
P78318	IGBP1	Immunoglobulin-binding protein 1	0.79	0.05	Inflammation	Signal transduction.
Q13724	MOGS	Mannosyl-oligosaccharide glucosidase	0.79	0.02	Metabolic process	N-glycan biosynthesis
Q5K4L6	SLC27A3	Solute carrier family 27 member 3	0.79	0.05	Metabolic process	Acyl-CoA ligase activity for long-chain and very-long-chain fatty acids
Q9Y285	FARSA	Phenylalanine--tRNA ligase alpha subunit	0.79	0.05	Metabolic process	Aminoacyl-TRNα biosynthesis
P53609	PGGT1B	Geranylgeranyl transferase type-1 subunit beta	0.78	0.03	Metabolic process	Transfer of a geranyl-geranyl moiety
Q13098	GPS1	COP9 signalosome complex subunit 1	0.78	0.04	Cell differentiation	Cop9 signalosome complex
Q6DD88	ATL3	Atlastin-3	0.78	0.03	Transport	Fusion of endoplasmic reticulum membrane
Q92688	ANP32B	Acidic leucine-rich nuclear phosphoprotein 32 family member B	0.78	0.01	Cell differentiation	Cell proliferation, apoptosis, cell cycle
Q96AT9	RPE	Ribulose-phosphate 3-epimerase	0.78	0.03	Metabolic process	Biosynthesis of amino acids
Q9NRY4	ARHGAP35	Rho GTPase-activating protein 35	0.78	0.02	Cell communication	Rho gap activity
Q9Y608	LRRFIP2	Leucine-rich repeat flightless-interacting protein 2	0.78	0.02	Metabolic process	Unknown
O60506	SYNCRIP	Heterogeneous nuclear ribonucleoprotein Q	0.77	0.03	Cell differentiation	mRNA processing mechanisms
Q13619	CUL4A	Cullin-4A	0.77	0.01	Metabolic process	Nucleotide excision repair
Q96JJ7	TMX3	Protein disulfide-isomerase TMX3	0.77	0.03	Metabolic process	Folding of proteins containing disulfide bonds
Q14444	CAPRIN1	Caprin-1	0.76	0.02	Cell communication	Synaptic plasticity in neurons and cell proliferation
Q9HCE1	MOV10	Helicase MOV-10	0.76	0.02	Metabolic process	mRNA target degradation
Q9NQX3	GPHN	Gephyrin	0.76	0.02	Cell structure	Membrane protein-cytoskeleton interactions
P05387	RPLP2	60S acidic ribosomal protein P2	0.75	0.01	Cell structure	Elongation step of protein synthesis.
Q14257	RCN2	Reticulocalbin-2	0.75	0.03	Metabolic process	Type 4 Bardet–Biedl syndrome
Q9BT40	INPP5K	Inositol polyphosphate 5-phosphatase K	0.75	0.04	Metabolic process	Insulin-dependent glucose uptake
Q9BV40	VAMP8	Vesicle-associated membrane protein 8	0.75	0.05	Inflammation	Platelet activation
Q9HCE6	ARHGEF10L	Rho guanine nucleotide exchange factor 10-like protein	0.75	0.04	Cell communication	Guanine nucleotide exchange factor
Q9P2M7	CGN	Cingulin	0.75	0.03	Cell-cell interaction	Tight junction
P23508	MCC	Colorectal mutant cancer protein	0.74	0.01	Cell death	Suppresses cell proliferation and the WNT/β-catenin pathway
Q9NR50	EIF2B3	Translation initiation factor eIF-2B subunit gamma	0.74	0.03	Metabolic process	RNA transport
Q06520	SULT2A1	Bile salt sulfotransferase	0.71	<0.01	Metabolic process	Bile secretion
P84090	ERH	Enhancer of rudimentary homolog	0.69	0.01	Metabolic process	Cell cycle
Q96B97	SH3KBP1	SH3 domain-containing kinase-binding protein 1	0.69	0.01	Cell-cell interaction	Endocytosis
O43306	ADCY6	Adenylate cyclase type 6	0.68	0.04	Metabolic process	Formation of the signaling molecule camp downstream of G protein-coupled receptors
P33176	KIF5B	Kinesin-1 heavy chain	0.68	0.04	Cell communication	Dopaminergic synapse, endocytosis
Q9NZ32	ACTR10	Actin-related protein 10	0.68	0.02	Cell structure	Microtubule-based movement
Q7L5Y1	ENOSF1	Mitochondrial enolase superfamily member 1	0.67	<0.01	Metabolic process	Fructose and mannose metabolism
Q9BQE3	TUBA1C	Tubulin alpha-1C chain	0.66	0.03	Cell structure	Constituent of microtubules
Q16611	BAK1	Bcl-2 homologous antagonist/killer	0.65	0.04	Cell death	Apoptosis
Q17RC7	EXOC3L4	Exocyst complex component 3-like protein 4	0.65	0.03	Transport	Unknown
Q9UNN5	FAF1	FAS-associated factor 1	0.65	0.03	Cell death	Ubiquitin-binding protein
Q9Y2W1	THRAP3	Thyroid hormone receptor-associated protein 3	0.65	0.02	Metabolic process	Pre-mRNA splicing
Q9H9C1	VIPAS39	Spermatogenesis-defective protein 39 homolog	0.64	0.03	Cell structure	Maintenance of the apical-basolateral polarity
Q96A49	SYAP1	Synapse-associated protein 1	0.62	0.03	Metabolic process	mTOTC2-mediated phosphorylation of AKT1
Q9BX59	TAPBPL	Tapasin-related protein	0.62	0.02	Inflammation	Antigen processing and presentation pathway,
P24386	CHM	Rab proteins geranylgeranyltransferase component A 1	0.58	0.04	Cell structure	Substrate-binding subunit of the Rab geranylgeranyltransferase complex
Q92539	LPIN2	Phosphatidate phosphatase LPIN2	0.58	0.02	Metabolic process	Metabolism of fatty acids
Q96AG3	SLC25A46	Solute carrier family 25 member 46	0.58	0.01	Cell structure	Mitochondrial organization
P02652	APOA2	Apolipoprotein A-II	0.57	0.02	Metabolic process	PPAR signaling pathway, stabilize HDL
Q9Y2U8	LEMD3	Inner nuclear membrane protein Man1	0.57	0.01	Cell communication	Repressor of TGF-β, activin, and BMP signaling
P02647	APOA1	Apolipoprotein A-I	0.55	0.02	Metabolic process	Reverse transport of cholesterol
P32456	GBP2	Guanylate-binding protein 2	0.50	0.05	Inflammation	NOD-like receptor signaling pathway
P13674	P4HA1	Prolyl 4-hydroxylase subunit alpha-1	0.47	0.03	Cell structure	Arginine and proline metabolism
Q9UKK3	PARP4	Protein mono-ADP-ribosyltransferase PARP4	0.47	0.03	Cell death	Apoptosis
Q8WWT9	SLC13A3	Solute carrier family 13 member 3	0.42	0.02	Transport	Sodium-coupled sulphate, di- and tri-carboxylate transporters

**Table 3:** The Suzuki histological criteria.

Grade	Congestion	Vacuolization	Necrosis
0	None	None	None
1	Minimal (10%)	Minimal (10%)	Singe-cell necrosis
2	Mild (<30%)	Mild (<30%)	Mild (<30%)
3	Moderate (30–60%)	Moderate (30–60%)	Moderate (30–60%)
4	Severe (>60%)	Severe (>60%)	Severe (>60%)

## Data Availability

Access to anonymized data will be granted upon reasonable request, on condition that researchers have appropriate ethical permission and sign the appropriate Material Transfer Agreement form.
